# Harnessing the Missing Spectral Correlation for Metasurface Inverse Design

**DOI:** 10.1002/advs.202308807

**Published:** 2024-07-01

**Authors:** Jie Zhang, Chao Qian, Guangfeng You, Tao Wang, Yasir Saifullah, Reza Abdi‐Ghaleh, Hongsheng Chen

**Affiliations:** ^1^ ZJU‐UIUC Institute, Interdisciplinary Center for Quantum Information State Key Laboratory of Extreme Photonics and Instrumentation Zhejiang University Hangzhou 310027 China; ^2^ ZJU‐Hangzhou Global Science and Technology Innovation Center Key Lab. of Advanced Micro/Nano Electronic Devices & Smart Systems of Zhejiang Zhejiang University Hangzhou 310027 China; ^3^ Jinhua Institute of Zhejiang University Zhejiang University Jinhua 321099 China; ^4^ State Key Laboratory of Integrated Service Networks Xidian University Xian 710071 China; ^5^ Department of Laser and Optical Engineering University of Bonab Bonab 5551395133 Iran

**Keywords:** bidirectional neural network, inverse design, metasurface, physical relation

## Abstract

A long‐held tenet in computer science asserts that the training of deep learning is analogous to an alchemical furnace, and its “black box” signature brings forth inexplicability. For electromagnetic metasurfaces, the related intelligent applications also get stuck into such a dilemma. Although the past 5 years have witnessed a proliferation of deep learning‐based works across complex photonic scenarios, they neglect the already existing but untapped physical laws. Here, the intrinsic correlation between the real and imaginary parts of the spectra are revealed using Kramers–Kronig relations, which is then mimicked by bidirectional information flow in neural network space. Such consideration harnesses the missing spectral connection to extract crucial features effectively. The bidirectional recurrent neural network is benchmarked in metasurface inverse design and compare it with a fully‐connected neural network, unidirectional recurrent neural network, and attention‐based transformer. Beyond the improved accuracy, the study examines the intermediate information products and physically explains why different network structures yield different performances. The work offers explicable perspectives to utilize physical information in the deep learning field and facilitates many data‐intensive research endeavors.

## Graphical abstract

Here, the intrinsic correlation between the real and imaginary parts of the spectra is revealed using Kramers–Kronig relations, which is then mimicked by bidirectional information flow in neural network space. The work offers explicable perspectives to utilize physical information in the deep learning field, and facilitates many data‐intensive research endeavors.

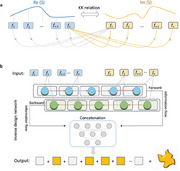

## 1 Introduction

Artificial electromagnetic media were born at the end of the twentieth century from the necessity of constructing arbitrary electromagnetic (EM) constitutive parameters to enrich the library of natural materials. Metasurfaces are one of the most thought‐provoking. Metasurfaces, propelled into the limelight after the wide acceptance of Snell's law,^[^ ^1^ ^]^ offer a compatible and planar design paradigm for manipulating EM waves at will. Due to the exceptional attributes of minimal thickness, enhanced integrability, and reduced insertion losses,^[^ ^2^, ^3^ ^]^ recent years have witnessed remarkable strides in the multitude of captivating metasurface applications, such as invisibility cloaks,^[^ ^4^, ^5^, ^6^, ^7^, ^8^, ^9^, ^10^, ^11^, ^12^ ^]^ high‐speed wireless communication,^[^ ^13^, ^14^, ^15^ ^]^ bioelectronics,^[^ ^16^ ^]^ quantum photonics,^[^ ^17^, ^18^ ^]^ and more.^[^ ^19^, ^20^, ^21^ ^]^

Central to metasurfaces is the fine control of nanostructure geometry and composition for a given electromagnetic characteristics (frequency, scattering, field enhancement and heat generation), making the study nearly impossible without computational techniques. In conventional approaches, the intricate relationship between metasurface structure and its electromagnetic response is often modeled through numerical simulations, exemplified by the finite‐difference time‐domain.^[^ ^22^ ^]^ However, for a given electromagnetic response, the inverse design necessitates extensive fine‐tuning of geometric configurations and case‐specific simulations, heavily relying on computational power and expert knowledge. However, the recent advancement of artificial intelligence has paved a clear path toward faster and more efficient design. The emergence of artificial intelligence‐assisted metasurfaces has revealed tremendous potential to revolutionize automatic design,^[^ ^23^, ^24^, ^25^, ^26^, ^27^ ^]^ customized devices,^[^ ^19^, ^28^, ^29^, ^30^, ^31^ ^]^ and self‐driven systems.^[^ ^32^, ^33^, ^34^ ^]^ Deep learning, as a formidable tool within the realm of artificial intelligence, has showcased unprecedented achievements across various domains, spanning computer vision,^[^ ^35^ ^]^ natural language processing,^[^ ^36^ ^]^ decision making,^[^ ^37^ ^]^ and more.^[^ ^38^, ^39^, ^40^ ^]^ With its data‐driven nature, deep learning possesses the ability to unearth intricate correlations within datasets, thereby revealing the implicit physics. In the context of metasurface inverse design,^[^ ^41^ ^]^ the integration of deep learning obviates the need for repetitive manual labor and time‐consuming simulations. Particularly in scenarios involving highly repetitive designs and real‐time applications where speed is of the essence, deep learning‐enabled inverse design methods exhibit significant prowess.

However, deep learning methods have long been regarded as an alchemical furnace, where the operation of the “black box” brings forth inexplicability.^[^ ^42^ ^]^ The physical interpretation and deep learning are like irreconcilable contradictions, with the former explaining phenomena in a lucid manner, while the latter exists without interpretability. In the realm of metasurface design, the selection of network structures largely relies on intuitive judgment. Many blind attempts are inevitable, and all attempts start from scratch when changing to new scenarios. In recent years, some studies have employed physical equations to assist in the reverse design of metasurfaces. However, most of these physical neural networks require designers to derive explicit physical formulas case‐by‐case, which increases the specificity and imposes higher demands on the designers' physical basis.^[^ ^43^, ^44^, ^45^, ^46^ ^]^ When utilizing existing architectures for reverse design, particularly in general scenarios, the reasons for why we choose this network and why this network performs better than another one, have always been overlooked. Besides, the physical connections in the input and output, as cheap additional items, have not been effectively utilized.

To address this challenge, we leverage the physical causality law between the real and imaginary parts of EM response, assisting in constructing a bidirectional information‐sharing mechanism. Utilizing this bidirectional sharing characteristic, we propose the utility of the bidirectional gated recurrent unit (GRU) network to extract and learn the complex interaction. By comparing unidirectional recurrent neural network (RNN), transformer structure based on attention mechanisms, and traditional fully connected neural network, we reveal the effectiveness of the bidirectional mechanism that aligns with the physical characteristics and improves performance by utilizing appropriate containers for feature extraction. Additionally, attempts to unveil the black box are made by peering into the effectiveness of different feature extractions through intermediate information products. A deeper understanding of the effectiveness of network architectures is gained here for metasurface inverse design. This physics‐inspired and semi‐open‐box design can revolutionize various fields, including metasurface engineering, photonics, optoelectronics, and material science, and provide a framework for universal reverse design, empowering further research and leading to breakthroughs in scientific and technological understandings.

## 2 Results

### 2.1 Revealing Spectral Connection Using Kramers–Kronig Relations

For any complex function *f*(ω) of a complex variable ω, the Kramers–Kronig relation^[^ ^47^ ^]^ can be written as follows,

(1) $$f\left(\omega \right)=u\left(\omega \right)+iv\left(\omega \right)$$

If $f\left(\omega \right)$ is analytic in the upper half‐plane, ω ∈ *R* ^+^ and $\lim_{|\omega |\to 0}f\left(\omega \right)\to 0$, here *R* ^+^ represents the set of positive real numbers, the Kramers–Kronig relation takes the form,

(2) $$u\left(\omega \right)=\frac{1}{\pi }PV\int_{-\infty }^{+\infty }\frac{v\left(\omega^{\prime }\right)}{\omega^{\prime }-\omega }\;d\omega^{\prime }$$

(3) $$v\left(\omega \right)=-\frac{1}{\pi }PV\int_{-\infty }^{+\infty }\frac{u\left(\omega^{\prime }\right)}{\omega^{\prime }-\omega }\;d\omega^{\prime }$$

Here, *PV* denotes the Cauchy principal value. It can be seen that the functions *u(ω)* and *v(ω)* are not independent; therefore, the complete function can be restored if only its real or imaginary part is given. For reflective metasurfaces, the reflective S‐parameter also termed as *R*, can be represented as *R* (ω) =  *Re*(*R*(ω)) + *iIm*(*R*(ω)), where *Re*(*R*(ω)) and *Im*(*R*(ω)) are real and imaginary parts, respectively. For example, under the short‐circuit conditions, the real part becomes *Re*(*R*(ω))  =  1, while *Im*(*R*(ω)) = 0. By introducing function *f(ω) = R*(ω) − 1, the following equations are derived:

(4) $$Re\left(R\left(\omega \right)\right)=\frac{1}{\pi }PV\int_{-\infty }^{+\infty }\frac{Im\left(R\left(\omega^{\prime }\right)\right)}{\omega^{\prime }-\omega }\;d\omega^{\prime }+1$$

(5) $$Im\left(R\left(\omega \right)\right)=-\frac{1}{\pi }PV\int_{-\infty }^{+\infty }\frac{Re\left(R\left(\omega^{\prime }\right)\right)-1}{\omega^{\prime }-\omega }\;d\omega^{\prime }$$

**Figure 1:**
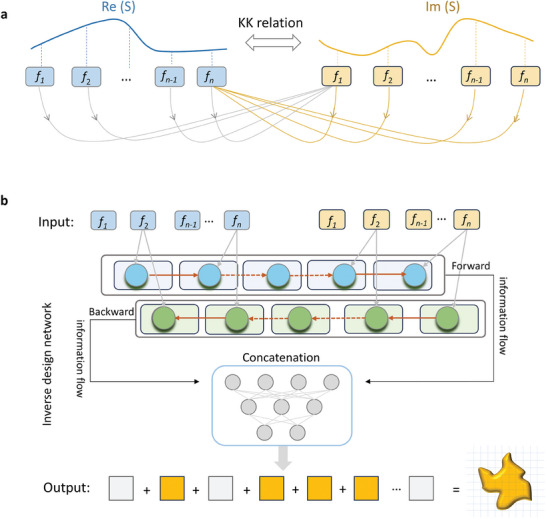
Revealing spectral connection for metasurface inverse design. a) The real and imaginary parts of the S‐parameter are coupled with each other based on the Kramers–Kronig relationship. b) For the inverse metasurface design, we aim to find proper metasurface candidates that can realize the target spectrum. To address the inverse design of the metasurface, we propose a scheme to enhance the performance of the inverse design network by leveraging the physical relationships between spectra. Inspired by this, a bidirectional recurrent training architecture is employed to mimic a rational flow of information and better extract the features of this information flow to enhance the network performance.

The above equations indicate that for a specific frequency point ω_0_, *Re*(*R*(ω_0_)) can be obtained from *Im*(*R*(ω)) at different frequency points, and vice versa, as shown in **Figure** **1**a. Other complex direct and indirect derivations of the relationship between real and imaginary parts of the reflective parameter can be found in Note S1 (Supporting Information). Drawing upon the aforementioned insights, it becomes apparent that the information pertaining to the real part plays a contributory role in shaping the information of the imaginary part, thereby implying a sense of directionality. Similarly, the S‐parameter information contained in the imaginary part exhibits a significant association with the individual frequency point information in the real part, thus indicating a directional flow of information. When using the RNN framework to process information, the input concatenates the real and imaginary parts, enabling the flow of information from the real to imaginary parts and vice versa.

For inverse design, we take a microwave resonant metasurface as an example, typically composed of a dielectric substrate (with the permittivity ε_*r*_, the period *p*, and the height *h*) and a metallic resonator. In Figure 1b, the metallic pattern discretized into a row of structural parameters is treated as a design target. In this scene, we concatenate the real and imaginary parts of the spectrum as input, similar to handling the influence between preceding and subsequent words in language translation. The real part of the S‐parameter at a specific frequency point is determined by the sequential integration of the frequency‐domain function containing the imaginary part of the S‐parameter at different frequency points, and vice versa. This implies that the sequential information of the imaginary part of the S‐parameter couples and influences the information of the real part. However, in practical scenarios, it is impossible to obtain the complete frequency spectrum with an infinite length. Within a limited frequency spectrum segment, we aim to leverage this underlying bidirectional information flow and employ a directional recurrent network to mimic it to capture the physical relationships and enhance the performance of inverse design by employing a rational flow of information (Re(S)→Im(S), Im(S)→Re(S)).

### 2.2 Mimicking Spectral Correlation Using the Bidirectional GRU Network

To highly mimic the physical correlation and bidirectional flow mechanism, we utilize a bidirectional GRU network to construct the inverse prediction network, aiming to better capture the characteristics between the real and imaginary parts. The GRU units^[^ ^48^ ^]^ are chosen because of their conciseness, allowing faster convergence during the training process. GRU units have the ability to quickly update and adapt their hidden state to capture relevant information in the current context. This adaptability enables them to effectively model sequential data and capture physical dependencies in the spectrum.

**Figure 2:**
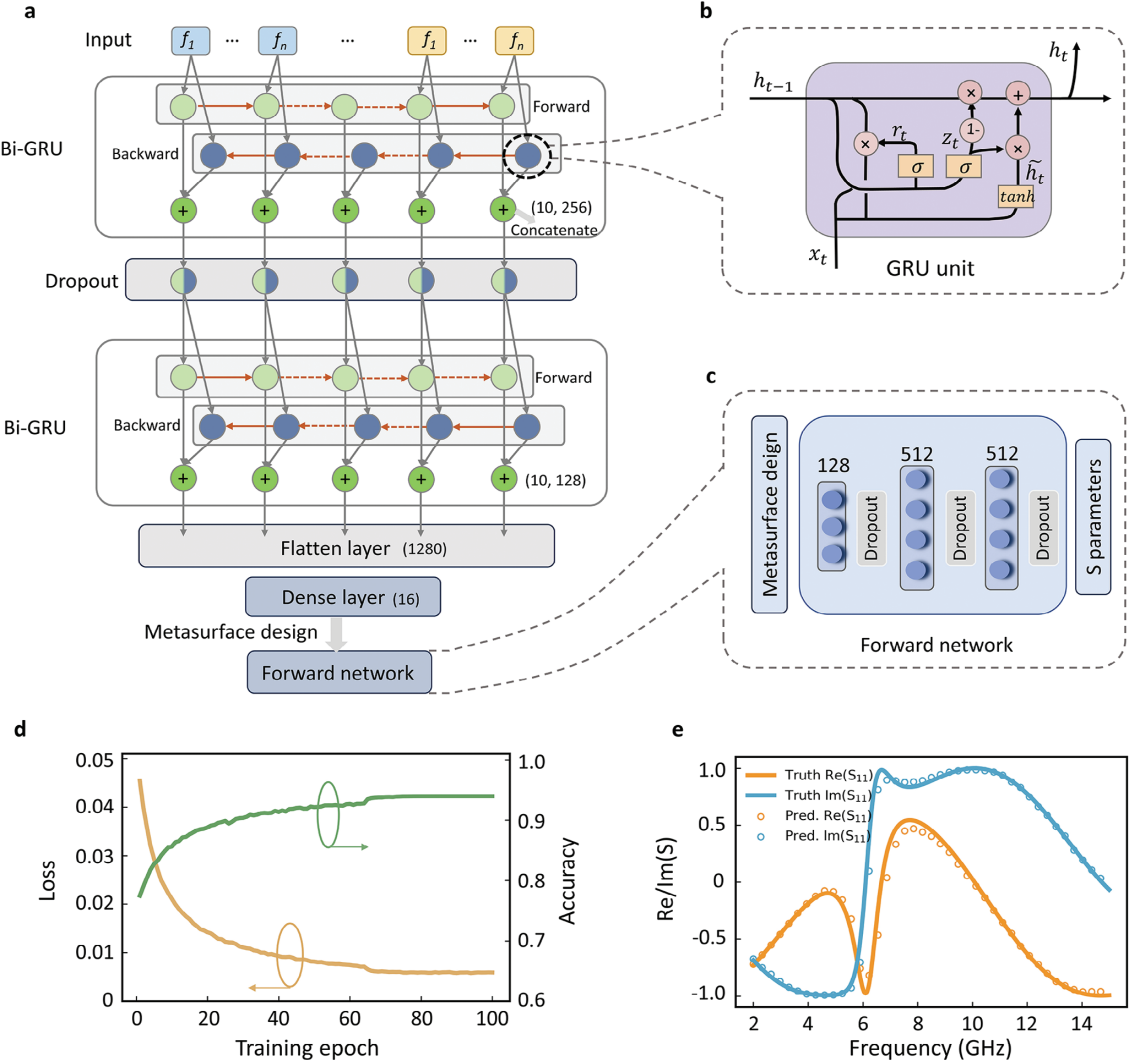
Bidirectional GRU inverse design network and training strategy. a) Architecture of the bidirectional recurrent neural network. The input layer divides the real and imaginary parts of the S‐parameter into (10200). The information is processed through two consecutive layers of bidirectional GRU units. Each bidirectional layer is formed by concatenating forward and backward neurons, enabling the bidirectional information flow and iteration. b) The internal structure of gated recurrent unit (GRU).c) For the tandem strategy, the forward problem is learned in advance and then fixed to train the inverse network. Tandem networks relax the requirements of converging, which alleviates the non‐uniqueness issue. d) The loss and the predicted accuracy of the forward network. e) Random test instance of the forward network.

In our work, the reflection spectra of interest are set from 2 to 15 GHz and uniformly spaced and discretized into 1000 real and 1000 imaginary data points. The specific network architecture, as depicted in **Figure** **2**a, is as follows: dividing the real and imaginary parts into 10 temporal steps, each of which comprises 200 frequency points. The information is then processed through two consecutive layers of bidirectional GRU units. Each bidirectional GRU layer is formed by concatenating forward and backward neurons, enabling the network to learn and integrate the physical information between the real and imaginary parts while extracting relevant features. After passing through the two bidirectional GRU layers, the neurons are flattened, and the flattened layer is mapped to a 16‐dff final output through a fully connected layer, thereby facilitating the inverse network. The design pattern is set as a vector with 16 dimensions. We choose the Adam optimizer, while the batch size is set to 100, the learning rate is set to 0.001. A function is defined to calculate the predicted accuracy, $P=\sqrt{\sum_{i=1}^{n}{\left(t_{i}-p_{i}\right)}^{2}}/\sqrt{\sum_{i=1}^{n}{\left(t_{i}\right)}^{2}}$, where *t_i_*, *p_i_*, and *n* denote the target reflection spectrum, the predicted spectrum, and the total amount of sampling points, respectively.

To relieve the non‐uniqueness issue, we adopt a tandem strategy.^[^ ^49^ ^]^ The inverse problem is partitioned into two sub‐problems, which are individually more amenable to fitting compared to the end‐to‐end training. At the initial step, the forward problem, a relatively straightforward task, is learned. This acquired knowledge of the underlying physics is subsequently employed in the second step to guide the training of the inverse network. During the joint training of the inverse network, the parameters of the pre‐trained forward surrogate network from the initial step are frozen, and both networks share the design space of structural parameters.

For the forward surrogate network, we employ a fully connected neural network to infer from metasurface structural parameters to the corresponding spectrum. The network architecture consists of neuron nodes with a configuration of 128‐512‐512, as depicted in Figure 2c. The loss and predicted accuracy are plotted in Figure 2d. During the training, we incorporate dynamic learning rate scheduling and implemented an early stopping strategy. Figure 2d demonstrates that, after 100 epochs of training, the model achieves a prediction accuracy of 94%. The randomly‐selected test case in Figure 2e demonstrates an excellent agreement between the predicted and target spectra. This highlights the superiority of the forward prediction network in accurately predicting the spectra, laying a solid foundation for inverse design in the subsequent stages.

### 2.3 Comparisons between Other Network Architectures

**Figure 3:**
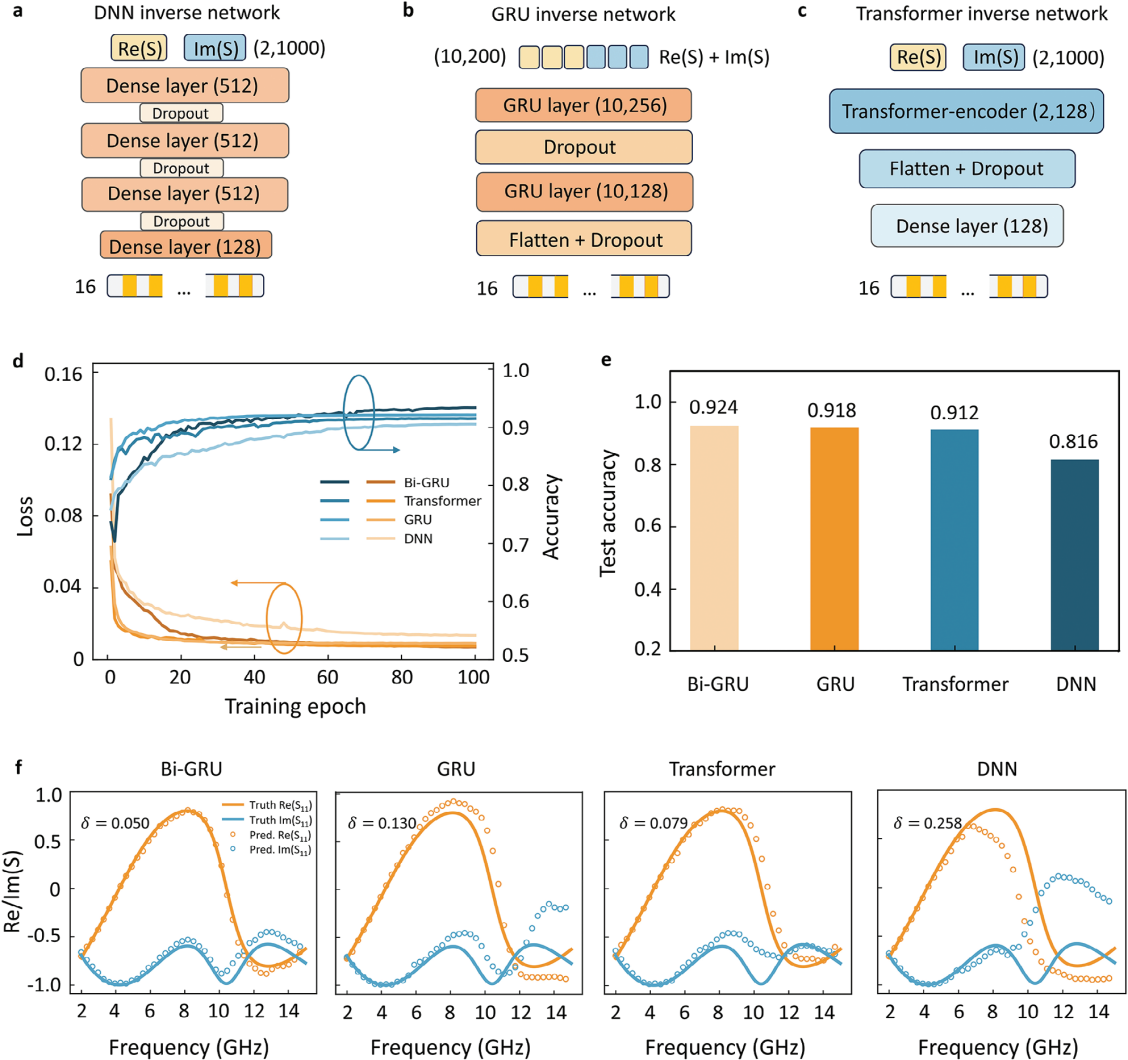
Comparison between other network architectures. a) DNN inverse network. b) Unidirectional GRU network. The input spectrum is reshaped to (10, 200). A two‐layer cascaded architecture with intermediate dropout layers is adopted, where each layer consists of 256 and 128 GRU units, respectively. c) Transformer inverse network. A transformer encoder is adopted to extract the information, whose hyperparameters are stated in the main text. Additional layers are incorporated, including a flattened layer, a dropout layer for regularization, and a dense layer for further processing. The network output is finally mapped to metasurface design feature space. d) The loss and predicted accuracy. e) The test accuracy of the Bi‐GRU, GRU, transformer, and DNN models. f) Test instances results from different architectures. The bidirectional GRU shows superior prediction performance.

To better highlight the feature extraction and learning capabilities of the bidirectional GRU structure, we also conduct a comparison with other classical architectures. To ensure the comparability of the control experiments, we use the same pre‐trained forward surrogate model in the joint training process. For the inverse design network, we utilize a fully connected neural network,^[^ ^50^ ^]^ unidirectional GRU, and transformer based on attention mechanisms.^[^ ^51^ ^]^ The architecture of the fully connected neural network is depicted in **Figure** **3**a. To highlight the performance enhancement achieved by the bidirectional mechanism, we conducted a comparison with a unidirectional GRU network. The architecture of the unidirectional GRU network closely resembled that of the bidirectional GRU network. We adopt a two‐layer cascaded architecture with intermediate dropout layers, where each layer consists of 256 and 128 GRU units, respectively. The resulting output is then flattened, followed by an additional dropout layer, and ultimately mapped to a 16D feature space. This specific construction of the unidirectional GRU network is illustrated in Figure 3b.

The transformer model is a successful and attention‐based model class known for its ability to prioritize important information and capture hierarchical correlations within input sequences. It has gained prominence in large‐scale language models. In the transformer architecture, the encoder and decoder share similar architectural components. The encoder utilizes multi‐head attention, while the decoder employs masked multi‐head attention mechanism, selectively masking out certain information. The masked mechanism in decoder prevents model from fully extracting features from bidirectional context information. In our study, we employed the transformer encoder architecture as a control group, reshaping the input into a (2, 1000) format to capture the interdependence between real and imaginary parts. The hyperparameters are set accordingly, the embedding dimension denoted as d_model is 128, the dimension of the feed‐forward layer called dff is 512, the number of attention heads is 8, and the maximum position encoding equals to 2000, which are depicted in Note S1 (Supporting Information). The transformer's attention mechanism is utilized to capture intricate dependencies and extract relevant features. Additional layers are incorporated, including a flattened layer, a dropout layer for regularization, and a dense layer for further processing. The output is mapped to a 16D space representing geometric structural parameters, encapsulating the acquired knowledge from the input data, as shown in Figure 3c.

In Figure 3d, we depict the variations in the loss (mean squared error, MSE) and the prediction accuracy, as defined by P, during the training process of models with different architectures. The whole dataset contains 10922 metasurfaces samples. The dataset was partitioned into training, testing, and validation sets following an 8:1:1 ratio. The loss curve signifies the convergence of the model during training, with lower values indicating better fitting to the target data, where the value of Bi‐GRU architecture indicated by the dark purple line is the minimum. The training prediction accuracy curve provides insights into the model's ability to produce the desired geometric structural parameters. The bidirectional GRU network depicted in the dark blue line outperforms other comparative networks in terms of prediction accuracy. When tested with the test data in Figure 3e, we find that the accuracies of the Bi‐GRU, GRU, transformer, and DNN models are 0.924, 0.918, 0.912, and 0.816, respectively.

We randomly select one case and compare the prediction results of different network structures under the tandem training strategy, focusing on the target input spectrum and the reverse‐designed predictions. The specific values of mean absolute error (MAE) represented by δ for each example are displayed in the top left corner of the images, as shown in Figure 3f. For these test instances, we find that the bidirectional GRU structure exhibited prediction results that were more in line with the target spectrum, specifically in the real and imaginary parts of the spectrum in high‐frequency region, compared to the DNN and unidirectional GRU structures. This suggests that the bidirectional GRU structure better captures the characteristics of the target spectrum in this scenario. These findings highlight the effectiveness of the bidirectional GRU structure in capturing the complex dependencies of the physical relationship of the spectrum. The bidirectional flow of information allows the network to incorporate both real and imaginary context, enabling it to better model and align with the target spectrum requirements. More test instances can be found in Note S3 (Supporting Information).

### 2.4 Visualizing Intermediate Information Products

**Figure 4:**
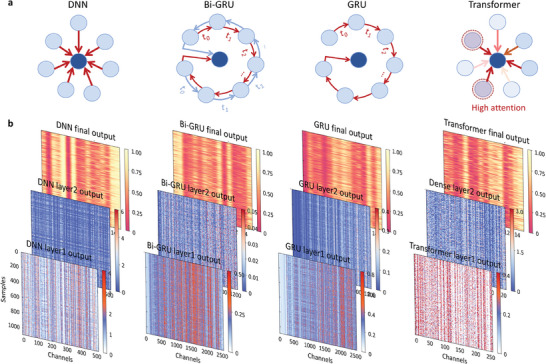
Visualizing intermediate information products. a) The connection mechanism of neurons for different architectures. b) The intermediate information variables displayed in the heatmap. The first two rows illustrate the values of intermediate information products from the first two layers in different inverse network inference processes. The x‐axis represents the channel numbers of the intermediate variables, while the y‐axis represents the test samples. Comparisons revealed that the features learned after the initial layer of the bidirectional GRU network displayed resemblances with the final 16D output and captured some extreme value characteristics. The outputs of the transformer structure exhibited faintly visible initial patterns but contained noise. Conversely, the second layer's intermediate variables in the underperforming fully connected network did not exhibit characteristic patterns approaching the final output.

To better compare the effectiveness of different networks in feature extraction, we reveal the learned intermediate information variables by depicting their changes. This process is intriguing as it differs from the conventional approach of directly obtaining output results. The black box is opened, aiming to explore the variations in information propagation. In **Figure** **4**a, the information interaction between the neurons of the upper layer and the neurons of the next layer is drawn under different architectures. For a fully connected neural network, the value of a neuron at the next layer is determined by the collective input from neurons in the previous layer. In contrast, for a bidirectional recurrent neural network, the new neuron information is jointly determined by the updated information from different directions after recurrent connections, where the information is concatenated. The critical distinction between unidirectional recurrent neural networks and bidirectional ones lies in the absence of bidirectional information interaction and fusion. As for transformer models based on attention mechanisms, attention scores are computed to determine the importance of different information to itself and other information. In the sequence output, higher attention scores assign greater attention and weight to information elements with higher importance. In Figure 4b, the first two rows illustrate the values of intermediate information products from the first two layers in different inverse network inference processes. Throughout the inference process, a total of 1092 sets of samples were utilized. The x‐axis represents the channel numbers of the intermediate variables, which may vary among different networks.

Through comparisons, we discover that after the first layer of the bidirectional GRU network, the learned features exhibited similarities with the final 16D output. Additionally, certain extreme value features have been captured, such as the patterns presented by the maximal value bands (highlighted in red) in the output of the first layer of the bidirectional GRU, and the 0 values (highlighted in red) in the 16D geometric output. In the output of the Transformer architecture, the red extreme values exhibit a faintly discernible pattern but still contain some noise, leading to a certain disparity with the final geometric output pattern. As for the relatively poorer performance of the fully connected network, there remains a significant gap between the extreme value pattern (red) of the first‐layer intermediate variables and the geometric feature pattern(red) of the final output. In our specific scenario, we observe that bidirectional information flow outperforms unidirectional recurrent learning. Besides, after undergoing feature extraction through the bidirectional GRU architecture, the structural similarity index (SSIM)^[^ ^52^ ^]^ (c1 = 0.1, c2 = 0.3, and c3 = c2/2) between the feature map layer and the predicted geometric parameter feature layer reaches 0.428. The SSIM value for the unidirectional GRU is 0.364, for the transformer architecture's feature map layer is 0.342, and for the DNN is 0.298. By concatenating the recurrent framework bidirectionally, the output information of specific real/imaginary position contains not only the potential correlations presented in one direction framework but also the synthesized information of all imaginary/real part signals carried by another direction framework within the frequency band. This result indicates more intuitively that through a single layer of bidirectional GRU mapping, the underlying complex correlations between spectra can be captured more quickly. Additionally, we find that the transformer‐based architecture does not demonstrate significant advantages in this context. The reason could be that attention‐based feature extraction relies on extensive data and large‐scale model dimensions. In the case of limited data and smaller model sizes, this advantage diminishes.

## 3 Experimental Verification

**Figure 5:**
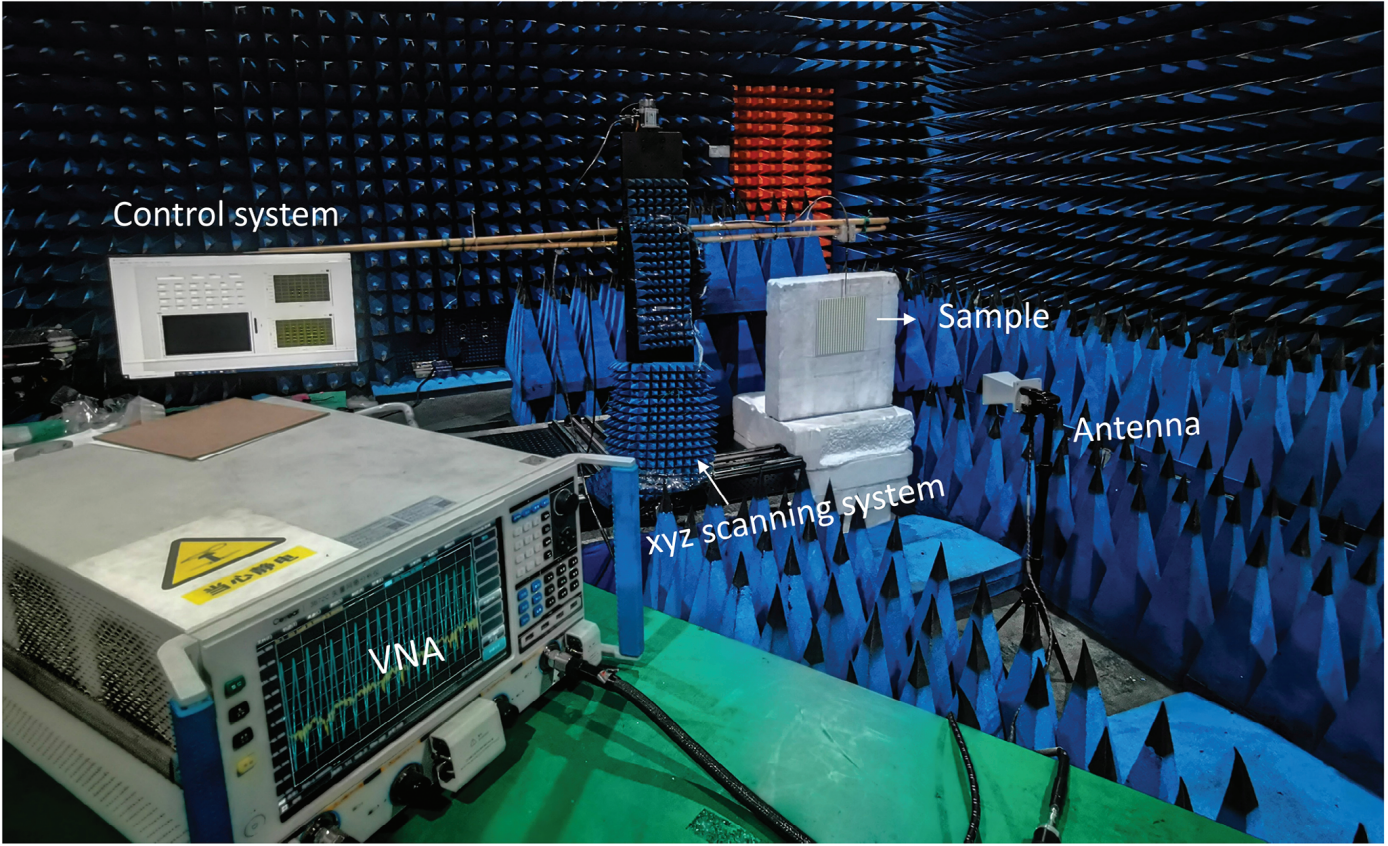
Measurement setup. The measurement setup consists of a transmitting antenna, a 3D measurement platform, a vector network analyzer, and a receiving antenna. The position of the receiving antenna can be flexibly adjusted using the control system, and the vector network analyzer's S_21_ parameter is utilized to collect the amplitude and phase information of the reflected parameter from the metasurface.

To experimentally verify our method, we fabricated and measured metasurfaces samples based on the geometric parameters predicted by Bi‐GRU. The measurement system consists of a transmitting antenna, a vector network analyzer (VNA), and a receiving antenna, as shown in **Figure** **5**. During testing, the transmitting antenna, receiving antenna, and metasurface sample are maintained at the same horizontal height. The receiving antenna is used to collect the amplitude and phase information of reflection parameter, which can be measured by the transmission coefficients (S_21_) obtained in VNA. The dimensions of the metasurface sample are 200 × 200 × 2mm3 (20 × 20 unit cells). Due to the approximation of finite unit cells of the metasurface, some deviations may occur in the high‐frequency region compared to the simulation data based on periodic boundary conditions. Therefore, we selected the low‐frequency range 4–8 GHz with relatively small approximation errors for experimental validation.

**Figure 6:**
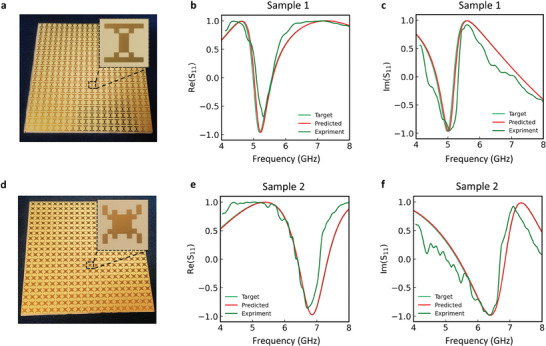
Metasurfaces prototype and measurement results. a,d) Sample 1 and sample 2 consisting of 20 × 20 units array metasurface, respectively. The insets are the enlarged version of the unit cell. b,e) Comparison of the real parts of the target, predicted, and experimental test reflection spectra of sample 1 and sample 2. c,f) Comparison of the imaginary parts of the target, predicted, and experimental test reflection spectra of sample 1 and sample 2.

**Figure** **6**a,d exhibits top geometric patterns produced through electroplating; see the enlarged figure in the insets of Figure 6a,d. The real and imaginary part of the reflection spectrum can be obtained by the S parameter of the sample which is calibrated and normalized using the denoised S parameter of the metallic reflector. The tested, predicted, and target spectrum, for the real and imaginary parts of the first sample are shown in Figure 6b,c. It can be observed that the main trends are consistent, but there is some inevitable oscillation. Figure 6e,f also present relatively good agreement of the experimental results for the second sample, thus validating the robustness of the method. However, there is some inconsistency between the experimental and theoretical simulation data, which could be attributed to the materials uncertainty and the approximation of the plane wave in the experiment.

## 4 Conclusion

In conclusion, an explicable perspective for deep learning to parse complex physical processes is showcased in metasurface inverse design. By leveraging the intrinsic physical correlation between the real and imaginary part of the spectrum and employing bidirectional information flow, we have demonstrated the ability to extract pertinent features more effectively. Our findings underscore the pivotal role of selecting an appropriate model architecture in augmenting the performance of inverse design networks. Comparative analysis with traditional deep neural networks, unidirectional networks, and transformer structures based on attention mechanisms has revealed the superior performance of bidirectional GRU networks in capturing intricate dependencies and accurately predicting the target spectrum. Efforts are undertaken to explore the effectiveness of various feature extractions by examining intermediate information products, thus attempting to unveil the inner workings of the black box. Additionally, we experimentally verified the robustness and effectiveness of the design method. It facilitates a more profound comprehension and utilization of the underlying physical principles and contributes valuable insights into the amalgamation of physics and artificial intelligence, offering practical guidance for selecting suitable network architectures.

## 5 Experimental Section

### Data Generation

The training data were obtained by co‐simulation performed using the commercial software Computer Simulation Technology (CST) Microwave Studio and MATLAB. First, MATLAB produced the pattern matrix ***R*** by uniformly sampling the complete solution space and transforming it into the corresponding binary matrix. The metasurface was discretized into a number of pixel blocks, represented as a matrix ***R***_*N* × *N*_, where *N* is the number of the discrete pixels of the resonator. ***R***_*N* × *N*_ is characterized by 1‐bit, where “1” signifies copper and “0” indicates vacuum, which are rendered as yellow and turquoise, respectively. In the case, this pattern can be encoded as *f*(*x*, *y*) ∈ {0, 1}^*N*^, where *x* and *y* are the coordinates of the metallic pattern. Here, we employed a set of four quadrant‐symmetric patterns and discretized each pattern in one quadrant into 4 × 4 pixels. The meta‐atom had a period of 10 mm with C4 symmetrical property. The meta‐atom consists of a three‐layer metal‐insulator‐metal configuration, where the intermediate dielectric substrate separates the bottom reflective surface from the top metal resonant pattern. The spacing substrate was a dielectric substrate F4B with a dielectric constant ε_*r*_ =  4.30, loss tangent *tan*δ  =  0.0025, and thickness *h*  =  2.0 mm. Second, the actual metasurfaces were imported into CST Microwave Studio to calculate the reflection coefficients. The reflection spectra of interest were set in the microwave region from 2 to 15 GHz and discretized into 1000 uniformly spaced real and imaginary data points. In this way, a total of 10922 metasurfaces were simulated. The dataset was partitioned into training, testing, and validation sets following an 8:1:1 ratio.

## Conflict of Interest

The authors declare no conflict of interest.

## Author Contributions

C.Q., J.Z., and H.C. conceived the idea of this research. J. Z. performed the network modeling and data analysis. J.Z. and C.Q. wrote the paper. R.A. checked and revised the paper. All authors shared their insights and contributed to discussions on the results. C.Q. and H.C. supervised the project.

## Supporting information

Supporting Information

## Data Availability

The data that support the findings of this study are available from the corresponding author upon reasonable request.
