# Supplementary material for: Harnessing the Missing Spectral Correlation for Metasurface Inverse Design
Source: Adv Sci (Weinh). 2024 Jul 1;11(33):2308807. doi: 10.1002/advs.202308807 (PMC11434224; doi:10.1002/advs.202308807)
Supplement: Supplementary file 1 — Supporting Information [file ADVS-11-2308807-s001.docx]

Supplementary information for

**Harnessing the missing connection in metasurface inverse design**

Jie Zhang^1,2,3^, Chao Qian^1,2,3,*^, Guangfeng You^1,2,3^, Tao Wang^4^, Yasir Saifullah^1,2,3^,

Reza Abdi-Ghaleh^5^, and Hongsheng Chen^1,2,3,*^

*^1^ZJU-UIUC Institute, Interdisciplinary Center for Quantum Information, State Key Laboratory of Extreme Photonics and Instrumentation, Zhejiang University, Hangzhou 310027, China.*

*^2^ZJU-Hangzhou Global Science and Technology Innovation Center, Key Lab. of Advanced Micro/Nano Electronic Devices & Smart Systems of Zhejiang, Zhejiang University, Hangzhou 310027, China.*

*^3^Jinhua Institute of Zhejiang University, Zhejiang University, Jinhua 321099, China.*

*^4^State Key Laboratory of Integrated Service Networks, Xidian University, Xian 710071, China.*

*^5^Department of Laser and Optical Engineering, University of Bonab, Bonab 5551395133, Iran.*

*^*^Corresponding auth*ors: chaoq@intl.zju.edu.cn (C. *Qian);* *hansomchen@zju.edu.cn (H. Chen)*

**Supplementary Note 1:** **The relationship between reflective parameters of spectrum**

In order to demonstrate the relationship of the reflective coefficients, we start from the simplified model. We assume a TM plane wave incident from air with permittivity $\varepsilon_{0}$ and permeability $\mu_{0}$ into another isotropic medium with permittivity $\varepsilon_{t}$ and permeability $\mu_{t}$.We assume the plane of incidence to be parallel to the x-z plane, which contains the incident wave vector and the surface normal. It can be derived from the boundary conditions of continuity of tangential $\bar{H}$ and $\bar{E}$ that:

$R=\frac{1-{\varepsilon_{0}k}_{tx}/\varepsilon_{t}k_{x}}{1+{\varepsilon_{0}k}_{tx}/\varepsilon_{t}k_{x}}$ (1)

For normal incidence, $k_{tx}=\omega\sqrt{\mu_{t}\varepsilon_{t}}$, $k_{x}=\omega\sqrt{\mu_{0}\varepsilon_{0}}$ . *R* can be simplified into$\frac{\sqrt{\varepsilon_{r}\left( \omega\right)}-1}{\sqrt{\varepsilon_{r}\left( \omega\right)}+1}$ ,where $\varepsilon_{r}\left( \omega\right)=\varepsilon_{t}(\omega)/\varepsilon_{0}$.

For instance, for a total short-circuit condition, $\varepsilon_{r}\left( \omega\right)$ can be equivalent to infinity to the whole frequency domain, leading to $\mathrm{Re}\left( R\left( \omega\right) \right)=1$, $Im\left( R\left( \omega\right) \right)=0$. For function $f\left( \omega\right)=Re\left( R\left( \omega\right) \right)-1+iIm(R\left( \omega\right)$, and $\lim_{\left| \omega\right|\to0} f\left( \omega\right)=0$. According to the Kramers-Kronig relation^[1]^, the following equation can be derived, where $PV$ denotes the Cauchy principal value,

$Re\left( R\left( \omega\right) \right)= \frac{1}{\pi}PV\int_{-\infty}^{+\infty} \frac{Im\left( R\left( \omega^{'} \right) \right)}{\omega^{'}-\omega} d\omega^{'}+1$ (2)

$Im\left( R\left( \omega\right) \right)= -\frac{1}{\pi}PV\int_{-\infty}^{+\infty} \frac{Re\left( R\left( \omega^{'} \right) \right)-1}{\omega^{'}-\omega} d\omega^{'}$ (3)

After discussing the extreme case, the cases of plasma-like materials will be introduced. For plasma media, $\varepsilon_{p}\left( \omega\right)=\varepsilon_{0}(1-\omega_{p}^{2}/\omega^{2})$, after substituting this expression into *R*, *R* can be written as:

$R\left( \omega\right)=\frac{-2\omega^{2}}{\omega_{p}^{2}}+1+2\omega\sqrt{\omega^{2}-\omega_{p}^{2}}$ (4)

when $\omega>\omega_{p}$, *R* is a real-valued function in the frequency domain. When $\omega<\omega_{p}$,$R\left( \omega\right)=\frac{-2\omega^{2}}{\omega_{p}^{2}}+1+i2\omega\sqrt{\omega_{p}^{2}-\omega^{2}}$, $R^{*}\left( \omega\right)=R(-\omega)$, and there is no point of divergence. When $\omega=\omega_{p}$, $R\left( \omega\right)=-1.$ This point is a special point, not a singularity (divergence). It's still resolvable. By constructing a function$f\left( \omega\right)=Re\left( R\left( \omega\right) \right)-1+iIm(R\left( \omega\right), \lim_{\left| \omega\right|\to0} f\left( \omega\right)=0$ is satisfied and $f\left( \omega\right)$ is analytic in the upper half-plane ***R***+. By applying Kramers-Kronig relation, the following equation can be derived,

$Re\left( R\left( \omega\right) \right)= \frac{1}{\pi}PV\int_{-\infty}^{+\infty} \frac{Im\left( R\left( \omega^{'} \right) \right)}{\omega^{'}-\omega} d\omega^{'}+1,$ (5)

$Im(R(\omega))= -\frac{1}{\pi}PV\int_{-\infty}^{+\infty} \frac{Re\left( R\left( \omega^{'} \right) \right)-1}{\omega^{'}-\omega} d\omega^{'}$ (6)

In addition, for more complex cases, the conditions under which the function of reflection parameters are fully analytic in the upper semi-complex plane remain to be investigated and need to be analyzed on a case-by-case basis. The relationship between the real and imaginary parts of the reflection parameter can be made to satisfy KK relationship by constructing reasonable functions.

Furthermore, we can construct the function in another way. $R\left( \omega\right)=r\left( \omega\right)e^{i\theta(\omega)}$, $\ln\left( R\left( \omega\right) \right)=\ln r\left( \omega\right)+i \theta(\omega)$. However, the function diverges logarithmically at infinity because $\lim_{\left| \omega\right|\to\infty} lnr\left( \omega\right)=O[ln(\omega)]$^[2]^. As a result, the function is not amenable to square integration over the real axis, and, consequently, simple dispersion relations and sum rules do not hold for $\ln r\left( \omega\right)$ and $\theta(\omega)$. The key to construction is a function involving $lnr\left( \omega^{'} \right)$ that approaches zero sufficiently rapidly at infinity for use in the Cauchy-theorem. Such a function is $f\left( \omega' \right)=\frac{\ln\left( R\left( \omega^{'} \right) \right)}{\omega^{'2}-\omega^{2}}.$

The function gives the well-known relation for the phase and the amplitude of complex reflectivity, as follows^[3]^:

$\theta\left( \omega\right)= \frac{-2\omega}{\pi}PV\int_{0}^{+\infty} \frac{lnr\left( \omega^{'} \right)}{\omega^{'2}-\omega^{2}} d\omega^{'}$ (7)

$Inr\left( \omega\right)-Inr\left( v \right)= \frac{2}{\pi}PV\int_{0}^{+\infty} \omega^{'}\theta\left( \omega^{'} \right)(\frac{1}{\omega^{'2}-\omega^{2}}-\frac{1}{\omega^{'2}-v^{2}}) d\omega^{'}$ (8)

By constructing functions to satisfy the integration conditions, the amplitude and phase relationships between the reflection parameters conform to a similar KK relation, revealing their bidirectional interaction and flow between the constructed real and imaginary parts. Through this indirect approach, the bidirectional information exchange between the real and imaginary parts of the reflection parameters can also be manifested.

**Supplementary Note 2:** **The architecture of transformer encoder**

To facilitate a comparative analysis of the attention-based transformer structure, we incorporated the encoder architecture of the transformer as a layer in the inverse design model. In the Transformer architecture, the encoder and decoder share similar architectural components. The encoder utilizes multi-head attention, while the decoder employs masked multi-head attention mechanism, selectively masking out certain information. The masked mechanism in decoder prevents model from fully extracting features from bidirectional context information. Therefore, we ultimately opted for the encoder module of the Transformer to learn attention between the real and imaginary components and perform further feature extraction. Figure S1a illustrates the detailed composition of the transformer encoder layer employed in the main text. Initially, the input information undergoes linear mapping to obtain embedding vectors, while simultaneously encoding positional information. The rules for positional encoding are defined by the following formula:

${PE}_{\left( pos,2i \right)=}sin(pos/{10000}^{2i/d})$ (9)

${PE}_{\left( pos,2i+1 \right)=}cos(pos/{10000}^{2i/d})$ (10)

where pos represents the position of a word in the sentence, d represents the dimension of the positional encoding (same as the embedding dimension d_model), 2i represents even dimensions, and 2i+1 represents odd dimensions (i.e., 2i ≤ d, 2i+1 ≤ d). The processed information vectors are then subjected to the operation rules of multi-head attention, resulting in intermediate vectors. After layer normalization, these intermediate vectors are added to the original embedding vectors and passed as input to the next layer. The specific operation mechanism of multi-head attention is depicted in Fig. S1c. By linearly mapping the embedding matrix shown in Fig. S1d, matrices Q, K and V are obtained. These matrices undergo the scaled dot-product attention operation, as shown in the Fig. S1e, to compute attention scores. The formula for calculation of attention is as follows:

$Attention(Q,K,V)=\mathrm{softmax}(\frac{QK^{T}}{\sqrt{d_{k}}})V$ (11)

where d_k_ refers to the dimensionality of the query and key vectors. The attention scores from multiple heads are concatenated, where the number of head is set as h here. Finally, the concatenated output is fed into a two-layer fully connected network with dff and d_model as the number of neurons,as illustrated in Fig. S1b. The output of the first fully connected neural network layer undergoes ReLU activation before entering the second layer, which does not require activation. After the operations of addition and normalization (Add and LayerNorm), the current layer's output is obtained.

**
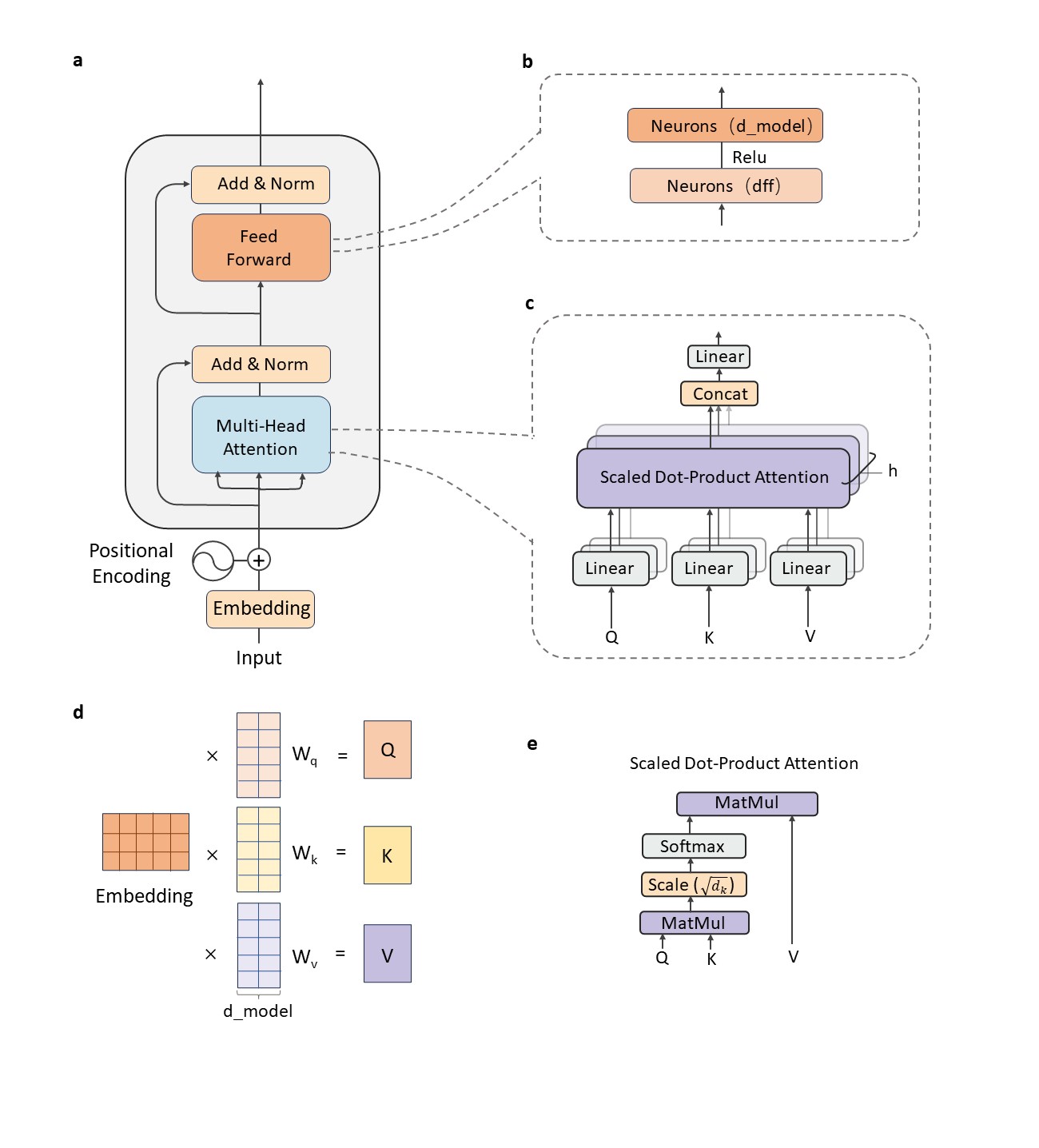
**.

**Fig. S1 | The detailed architecture of transformer encoder**. **a**, The internal structure of the transformer encoder is as follows: The input information is firstly passed through an embedding layer, and positional encoding information is added. The resulting vectors are then subjected to multi-head attention computation. After undergoing Add and Norm operations, the vectors enter a two-layer fully connected network. The output is processed in the same manner. **b**, The architecture of the two-layer fully connected network consists of multiple neural nodes, with specific numbers as dff, d_model. **c**, The computation rules for multi-head attention involve calculating the values of attention based on scaled Dot-Product Attention mechanism and following the architecture. **d**, The embedding vectors undergo linear mapping to generate Q, K, and V vectors. **e**, The scaled dot-product attention operation calculates the attention values for the Q, K, and V matrices according to the above process.

**Supplementary Note 3:** **Test instances**

We conducted a random selection of another two cases and analyzed the prediction outcomes of different network architectures. Our focus was on the target input spectrum and the predictions generated through reverse design. The mean absolute error (MAE) values for each example are presented in the top left corner of the corresponding images, as depicted as$\delta$. In these two test instances shown in Fig.S2, it was observed that the bidirectional GRU architecture yielded prediction results that closely matched the target spectrum, particularly in the real and imaginary parts of the spectrum within the high-frequency range. In comparison, the DNN and unidirectional GRU structures exhibited less accuracy in capturing the characteristics of the target spectrum. To better illustrate the differences between different networks, we also present additional spectral curves with more rapid changes, as shown in Fig.S3. In the spectral examples with rapid changing features, we observed that the bidirectional GRU network outperforms other comparative networks not only in predicting flat spectra but also in predicting spectra with rapidly changing characteristics. These findings suggest that the bidirectional GRU architecture effectively captures the relevant features of the target spectrum in this particular scenario.


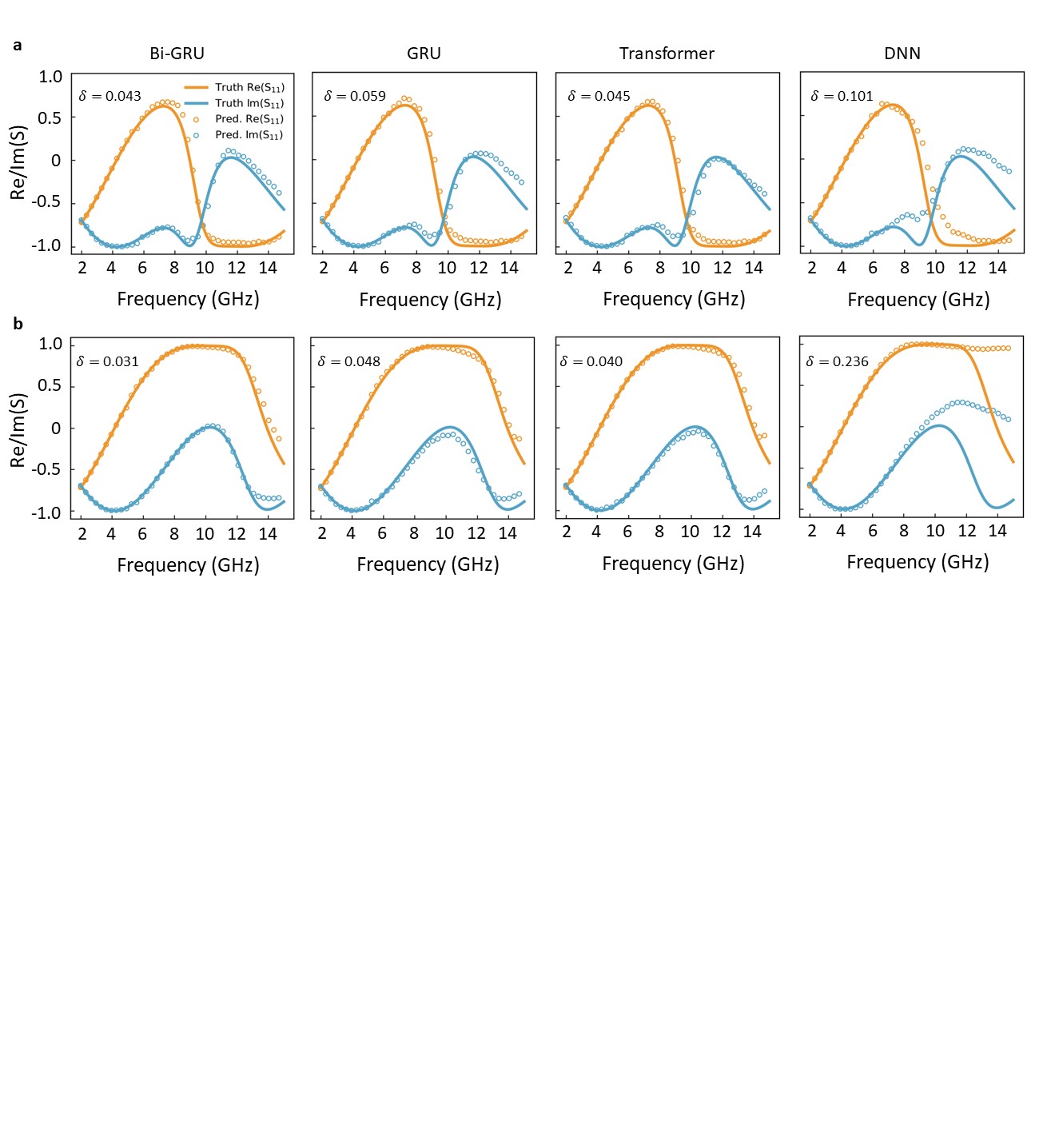


**Fig. S2 | Test instances. a**, **b**, The results obtained from various architectures in the test instances revealed that the bidirectional GRU architecture exhibited superior predictive performance in relation to the target spectrum, surpassing the performance of the DNN, unidirectional GRU, and transformer architectures.


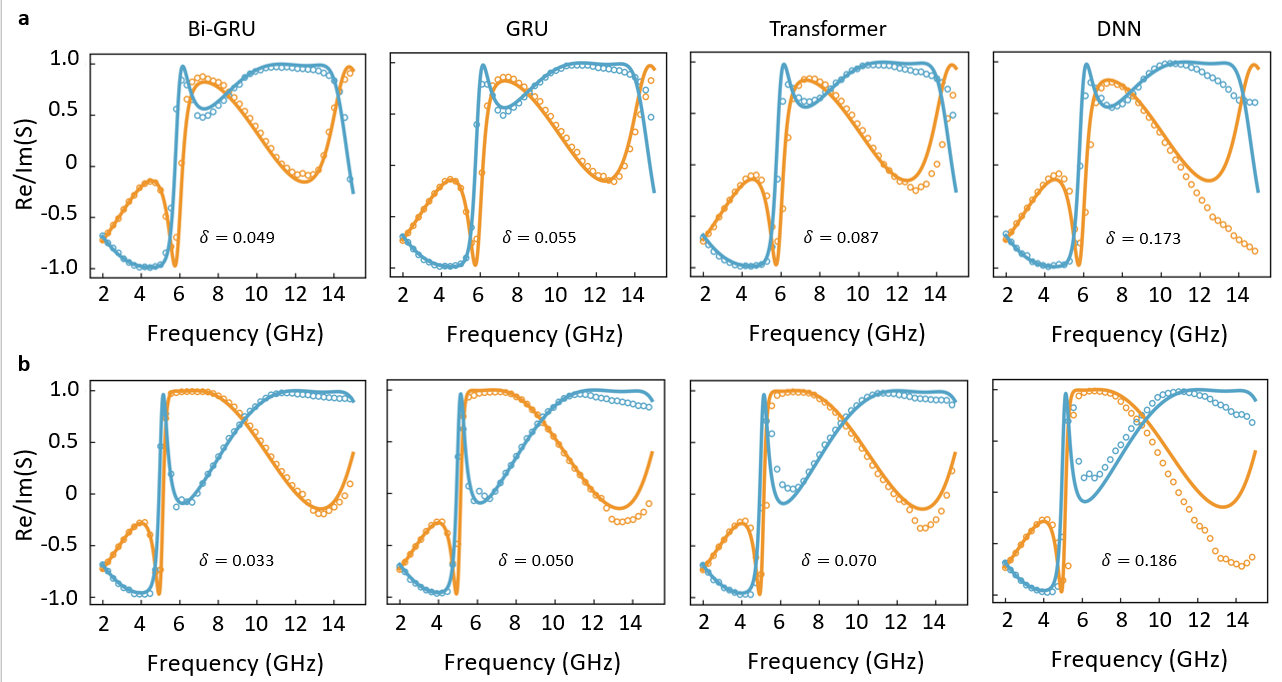


**Fig. S3 | Test instances. a**, **b**, The results obtained from various architectures in the test instances revealed that the bidirectional GRU architecture exhibited superior predictive performance in relation to the target spectrum, surpassing the performance of the DNN, unidirectional GRU, and transformer architectures.

**References**

[1] Staelin D. H., Morgenthaler A. W. & Kong J. A. *Electromagnetic waves*. Pearson Education India (1994).

[2] Smith D. & Manogue C. A. Superconvergence relations and sum rules for reflection spectroscopy. *J. Opt. Soc. Am.* **71**, 935-947 (1981).

[3] Smith D. Y. Dispersion relations for complex reflectivities. *J. Opt. Soc. Am.*  **67**, 570-571 (1977).
